# I don’t care why you do it, just don’t! Reactions to negative and positive organizational deviance partly depend on the desire for tightness of prevention-focused employees

**DOI:** 10.3389/fpsyg.2022.951852

**Published:** 2022-10-06

**Authors:** Silvana Mula, Antonio Pierro

**Affiliations:** Department of Developmental and Social Psychology, Sapienza University of Rome, Rome, Italy

**Keywords:** tightness–looseness, desire for tightness, regulatory prevention focus, norm-violation, organizational deviance

## Abstract

Tightness–Looseness (T-L) at the individual level has only begun to receive attention from researchers. Specifically in the organizational context, this is a so far unexplored construct. The study offers first insights into the mechanisms that can trigger individuals’ desire for tightness and the consequences it can have on organizational behaviors. We, therefore, investigated the mediating role of the desire for tightness on the relationship between work regulatory prevention focus and emotional responses to both negative and positive (i.e., pro-social) deviant organizational behaviors. We tested our prediction through a mediational model with a sample of 788 Italian employees (58.6% females, *M*age = 35.09). Our findings supported the hypothesized model showing that regardless of the motivation underpinning the norm-violating behavior, employees with a prevention focus are more desirous of tightness and exhibit hostile reactions toward deviance. Given its importance in understanding employees’ behaviors and intentions, which inevitably reflect on the organization’s functionality, the impact of the T-L individual-level dimension in organizations is undoubtedly worthy of deeper investigation.

## Introduction

Clear and well-defined norms, respect for them, and the consequent lack of deviant behaviors are deemed the basis for well-functioning communities, including organizations. A cardinal construct to give a key to understanding this perspective is that of cultural tightness–looseness (T-L) (e.g., Gelfand et al., 2006), which can be defined as a continuum combining the level of strength of social norms with tolerance for norm deviance. The strength of social norms refers to both unwritten and institutionalized rules that exist within societies or communities as well as the degree of social pressure that individuals feel to respect them, while tolerance for norm deviance denotes the amount of penalties provided when those norms are violated.

The cultural dimension of T-L can have top-down and bottom-up influences at individual, societal and organizational levels (Gelfand et al., 2006). For example, T-L at societal-level affects people’s dispositional attributes,– when compared to persons in looser cultures, those in tighter cultures show higher prevention focus, impulse control, self-monitoring, and need for structure (Gelfand et al., 2011; Harrington and Gelfand, 2014). The inverse is also true, so that individuals’ personal characteristics can move the external environment in a tight or loose way. For example, individuals with higher felt accountability (e.g., a regulatory prevention focus, high regulatory strength, etc.), typical of tight societies, are more likely to develop and sustain shared norms that prioritize order, control, discipline, and conformity (Gelfand et al., 2006).

To date, T-L has received only sporadic attention in the organizational culture literature, with much of the theory and research focusing nearly entirely on its impact at the group level of analysis (Crossland and Hambrick, 2011; Toh and Leonardelli, 2012; Ozeren et al., 2013; Peltokorpi and Froese, 2014; Chua et al., 2015; Aktas et al., 2016; Huang and Ren, 2017; Kim and Toh, 2019; Gedik and Ozbek, 2020; Di Santo et al., 2021). In the light of the current literature, there seems to emerge a gap of individual level (i.e., individuals’ desire) research on cultural T-L. While group-level measures of T-L depict people’s shared perception of the existence of clear and well-defined norms in their society, country, and/or workplaces, individual-level measures reflect one’s subjective view of how strict these norms should be in their living (or working) context and how much the latter should be intransigent toward deviance. Although the two constructs are distinct, T-L at the group level (i.e., shared tightness–looseness) and T-L at the individual level (i.e., supported or desired tightness–looseness) are theorized to be associated with the same correlates (see Jackson et al., 2019). Empirical evidence for this perspective derives from recent studies. For example, perceived threat activates both the shared perceived (group level) (Jackson et al., 2019), and the supported and desired (individual level) tightness (Jackson et al., 2019; Mula et al., 2021, 2022; Baldner et al., 2022; Stamkou et al., 2022). T-L was also found to be associated with prejudices and negative attitudes toward marginalized groups at both group level (Jackson et al., 2019) and individual level (Jackson et al., 2019; Mula et al., 2022), and with self-control and impulses controlled at both the group (Gelfand et al., 2011; Harrington and Gelfand, 2014; Mu et al., 2015) and individual level (Mula et al., 2021).

In the organizational context, T-L at the individual level remains an unexplored construct. We seek to begin to fulfill this void by investigating the mechanisms that can trigger employees’ desire for cultural tightness and the consequences it can have on their organizational behavior. Noteworthy, it is of fundamental importance to study and deepen the intrapersonal and interpersonal dynamics within organizations: How people approach the norms, how they set themselves toward the goals to be achieved, and how they relate to others. It is in fact the attitudes, behaviors, and desires of workers that can push the organization toward well-being or, on the contrary, toward malaise. Therefore, the present research aims to examine if individuals’ motivational principles (i.e., regulatory prevention focus) may spark their desire for tightness and if this latter could have an impact on the responses toward deviant behaviors. Specifically, we assumed that employees with a work prevention focus should wish more tightness in their workplaces and this, in turn, should be associated with their emotional reactions toward deviant organizational behaviors.

### Prevention focus, desire for tightness, and emotional reactions to deviance

Following regulatory focus theory (e.g., Higgins, 1998, 2012), people’s attitudes, behaviors, intentions, and emotional reactions are influenced by their predominant—prevention vs. promotion—regulatory focus. While promotion-focused self-regulation is concerned with attaining growth and accomplishment, prevention-focused self-regulation is mainly concerned with satisfying duties, responsibilities, and obligations, and it is motivated by security and safety by adhering to standards and rules. Specifically, when a person is prevention-focused, for example, he or she seeks to avoid behaviors that are inconsistent with a goal or norm (Higgins, 1997, 1998). Indeed, given their concern with duties and responsibilities (“*oughts*”), individuals with a prevention focus generally react strongly (i.e., with anger) to norm violation (Keller et al., 2008). Thus, prevention-focused individuals are likely not only to avoid engaging in deviant behaviors, but they may also be less tolerant of norm-violating behaviors. Against this backdrop, it is reasonable supposing that employees guided by a prevention-focused at work would be more desirous of tightness and this, in turn, would elicit their negative responses toward organizational deviance. From an organizational standpoint, “organizational deviance” is defined as “voluntary behavior that violates significant organizational norms and in so doing threatens the well-being of an organization, its members, or both” (Robinson and Bennett, 1995, p. 556). It encompasses a wide range of counterproductive work behaviors, including stealing property from work without permission, taking unexpected breaks, and failing to follow supervisors’ instructions. In addition to this “negative” organizational deviance, recent studies have begun to recognize that norm-deviating behaviors might assume a “positive” value when they are aimed at benefiting the organization itself and the welfare of stakeholders (Dahling et al., 2012; Bashshur and Oc, 2015). In this respect, tight cultures seem to be intransigent toward deviance regardless of whether the motivations behind the norm-violating behavior are negative or positive (Gelfand et al., 2011). In fact, if on the one hand, tight cultures condemn forms of negative deviance, sustaining severe sanctions against those who deviate (e.g., death penalty), on the other hand, tight societies also condemn forms of positive deviance, such as protesting for one’s own and others’ rights (e.g., signing petitions, going on strike). We thus decided to seek support for this argument by investigating employees’ emotional reactions to both negative and positive organizational deviant behaviors. More in particular, we investigated responses to pro-social organizational rule-breaking, which is intended as a form of positive deviance “characterized by volitional rule-breaking in the interest of the organization or its stakeholders” (Dahling et al., 2012, p. 21).

### The present study

In the current study, we tested the mediational effect of Italian employees’ desire for tightness on the relationship between work prevention focus and reactions to workplace deviant behaviors, controlling for employees’ age, gender, educational level, and seniority. More specifically, we hypothesize that regulatory work prevention focus is positively associated with high desire for tightness which, in turn, is positively related with emotional reactions to both positive and negative organizational deviance. The proposed model was tested using the Process Macro for SPSS (Hayes, 2018), applying Model 4 with 5,000 bootstrap samples. We performed two independent mediation models, one using reactions to deviant organizational behavior as the outcome variable and the other with reactions to pro-social workplace deviance as the outcome variable.

## Materials and methods

### Participants

Eight hundred eighty-four employees in Italian organizations were recruited through Prolific Academic, an online participant recruitment platform, and received monetary compensation for participating in a cross-sectional online survey. Sixty participants left the survey entirely blank and were excluded from the analysis, as well as students (*N* = 19), unemployed (*N* = 4), and freelancers (*N* = 13). The final sample consisted of 788 Italian employees. Ages ranged from 18 to 69 (*M* = 35.09, *SD* = 11.47). Most participants were women (58.6%); 0.4% reported having a primary education, 34% possessed a higher school diploma, 19% had a bachelor’s degree, 31.3% had a master’s degree, and 13.8% had a higher education (e.g., Ph.D.). Participants also indicated their seniority (*M* = 8.17, *SD* = 9.34) and type of employment. All participants worked together with colleagues and worked either in public (e.g., schools, police departments, post-offices, etc.) or private organizations (e.g., no-profit organizations, manufacturing organizations, etc.). After giving their informed consent, participants completed the following measures. All study materials were presented in Italian.

## Measures

### Work prevention focus

Employees’ work prevention focus was assessed with the 9-item prevention subscale from the Work Regulatory Focus Scale developed by Neubert et al. (2008) (e.g., “I am very careful to avoid exposing myself to potential losses at work”). The Cronbach’s *α* for the prevention scale was *α* = 0.85.

### Desire for cultural tightness

We measured desire for tightness through five items from Gelfand et al. (2011) and previously used in recent studies (Mula et al., 2021; Baldner et al., 2022; Di Santo et al., 2022), adapted to the organizational context. Participants were asked to indicate to what extent the organization they currently work in should have loose versus tight characteristics (e.g., “Being tolerant of those who violate the rules” vs. “Being intransigent with those who violate the rules”; “Having flexible social norms” vs. “Having rigid social norms”). Each item was responded to on a 9-point scale. Higher values reflect greater desire for tightness. The scale had acceptable internal consistency (Cronbach’s *α* = 0.74).

### Reaction to workplace deviance

#### Reaction to organizational deviant behaviors

Reactions to workplace deviant behaviors were assessed with 12 items from the Organizational Deviance subscale of the Interpersonal and Organizational Deviance Scale developed by Robinson and Bennett (1995). Participants were asked to read a list of behaviors that can occur at work (e.g., “Taken property from work without permission”). They had to rate what would be their most likely emotional reaction if they found someone engaging in such behaviors in the workplace (from 1 = Approval to 5 = Violent fury). The Cronbach’s *α* for this scale was acceptable (*α* = 0.92*)*.

#### Reaction to pro-social work deviance

Employees indicated their most likely emotional reaction (from 1 = Approval to 5 = Violent fury) to pro-social work deviance (e.g., “Breaking organizational rules to provide better customer service”) through three items adapted from Dahling et al. (2012). The scale had acceptable internal consistency (Cronbach’s *α* = 0.84).

## Results

Descriptive statistics and correlations between variables are presented in Table 1. As can be seen, work prevention focus was positively and significantly related to desire for tightness (*r* = 0.24, *p* < 0.001) as well as to both reactions to organizational deviant behaviors (*r* = 0.28, *p* < 0.001) and the reactions to pro-social work deviance (*r* = 0.20, *p* < 0.001). Moreover, desire for tightness was positively and significantly associated with negative reactions to organizational deviant behaviors (*r* = 0.33, *p* < 0.001) and the reactions to pro-social work deviance (*r* = 0.28, *p* < 0.001).

**Table 1:** Descriptive statistics and correlations between variables.

	1	2	3	4	5	6	7	8	*M (SD)*
1. Work prevention focus	–								5.08 (0.63)
2. Desire for tightness	0.24^**^	–							5.32 (1.38)
3. Reactions to negative organizational deviance	0.28^**^	0.33^**^	–						3.25 (0.65)
4. Reactions to positive organizational deviance	0.28^**^	0.28^**^	0.61^**^	–					2.58 (1.02)
5. Age	0.16^**^	0.26^**^	0.37^**^	0.23^**^	–				35.09 (11.05)
6. Gender	0.15^**^	0.04	0.14^**^	0.11^*^	0.15^**^	–			–
7. Education	−0.15^**^	−0.12^*^	−0.03	−0.03	−0.01	0.19^**^	–		–
8. Seniority	0.18^**^	0.25^**^	0.34^**^	0.18^**^	0.84^***^	0.09^*^	−0.12^*^	–	8.17 (9.34)

*p < 0.01; **p < 0.001.

### Mediation analysis

**Figure 1:**
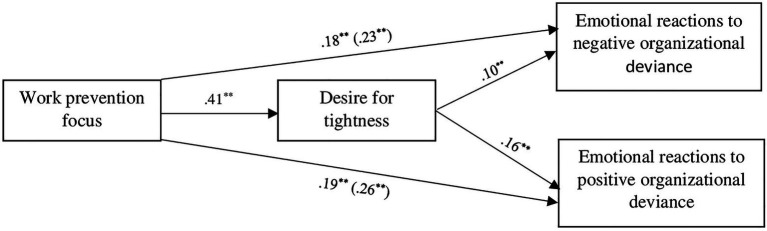
Effect of work prevention focus on reactions toward negative and positive organizational deviance *via* desire for tightness. *N* = 788 All coefficients are unstandardized. The total effect is inside the parentheses ^*^p < 0.01, ^**^p < 0.001. High s of reaction toward organizational deviance reflects higher disapproval (i.e., anger, fury).

The results of the mediation models are summarized in Figure 1. The first mediation revealed a significant and positive effect of work prevention focus on desire for tightness (*b* = 0.41, *t* = 5.41, *p* < 0.001 95% CI [0.265, 0.567]), which, in turn, had a significant and positive effect on reactions to organizational deviant behaviors (*b* = 0.10, *t* = 6.32, *p* < 0.001 95% CI [0.069, 0.130]). The total effect of work prevention focus on reactions to organizational deviant behaviors was significant (*b* = 0.23, *t* = 6.51, *p* < 0.001, 95% CI [0.157, 0.293]), as well as the direct effect (*b* = 0.18, *t* = 5.34, *p* < 0.001 95% CI [0.116, 0.252]). More importantly, the analysis revealed a significant indirect effect of work prevention focus on reactions to organizational deviant behaviors *via* desired tightness (*b* = 0.04, 95% CI [0.022, 0.064]), confirming our hypothesis of an at least partial mediating role of desired tightness on the relationship between prevention focus and hostile reactions to organizational deviance.

The second mediation showed significant and positive effect of desire for tightness on reactions to pro-social work deviance (*b* = 0.16, *t* = 5.98, *p* < 0.001 95% CI [0.106, 0.210]). The total effect of work prevention focus on reactions to pro-social work deviance was significant (*b* = 0.26, *t* = 4.53, *p* < 0.001 95% CI [0.149, 0.376]), as well as the direct effect (*b* = 0.19, *t* = 3.41, *p* < 0.001 95% CI [0.084, 0.310]), Notably, once again and confirming our hypothesis, we found a significant indirect effect of work prevention focus on reactions to pro-social work deviance through desire for tightness (*b* = 0.07, 95% CI [0.035, 0.102]).

All results were obtained by controlling for employees’ age, gender, educational level, and seniority.

## Discussion

Consistent with our hypotheses, we found that employees with a strong work prevention focus have greater desire for tightness which, in turn, is reflected in a lower tolerance for workplace deviant behavior. In this vein, employees who carefully avoid potential failures and losses at work also generally wish their organization to have more restrictive rules and to be uncompromising toward those who do not respect these rules. This desire, for its part, is mirrored in adverse emotional reaction (i.e., anger) to deviant behaviors at work, whether aimed at harming or benefiting the organization itself. These noteworthy results show that for prevention-focused employees, eager for tightness, there seems to be no justification for engaging in deviant behaviors—they will respond with anger and disapproval even when deviant behavior is enacted in order to give an advantage to the organization. Certainly, this is the first empirical evidence that will need further investigation in future research.

Nevertheless, these findings should make a remarkable contribution to the field of the cultural tightness–looseness body of research. This is the first study to investigate the variables that can trigger and be triggered by the desire for tightness in the workplace. In this way, we further advance empirical understanding of the relationship between prevention regulatory focus and desired tightness, compared to previous studies that have only studied the extent to which cultural tightness is associated with prevention’s correlates (i.e., conscientiousness and dutifulness). In fact, although a link between prevention focus (i.e., focus on avoiding negative outcomes) and T-L has been proposed (Gelfand et al., 2006), Gelfand et al. (2011) and Harrington and Gelfand (2014) empirically looked at the relationship between state and national-level T-L and conscientiousness and dutifulness, finding that individuals in tight states and in tight nations tended to behave appropriately and avoid mistakes.

Moreover, even though the notion of cultural tightness already implies a low degree of tolerance toward norm-violating behaviors (Gelfand et al., 2011; Gelfand, 2018), this study provides a first attempt to empirically prove the association between the desire for tightness and negative emotional reactions toward both positive and negative deviant behaviors. Our findings overall provide support to previous results from Kim and Toh (2019), which explored the influence of leader’s past experience with organizational tightness on the frequency of both positive and negative deviant behaviors of the group. In addition, our findings revealed a direct effect of work prevention focus on hostile reactions to organizational deviance. It is thus important to remember that responses to norm-deviating behaviors may be influenced by more than just desired tightness. In fact, regardless of their need for stringent norms and strong sanctions, prevention-focused employees might directly disapprove both positive misbehavior and negative misbehavior. Future studies should explore other possible mediators of the relationship between work prevention focus and adverse reactions toward deviance.

Despite its novelty, our study is not without limitations. The first limitation concerns the cross-sectional nature of our research design. In fact, self-reported data without experimental manipulations do not allow to deduce causal relationships between the examined variables.

Moreover, although the proposed mediating effect of desired tightness on relationships between work prevention focus and responses to deviant organizational behaviors was shown to have overall support, we only examined desired T–L without taking into account the actual tightness or looseness of the organizational culture. Following Gelfand et al. (2006), the tightness–looseness of an organization depends on the tightness–looseness of both the national culture in which the organization is placed and on the personal level of tightness–looseness of the individuals. In this regard, given that our sample was from Italy, which is commonly regarded as having a slightly loose culture (Gelfand et al., 2011), the Italian organizations in which our recruited employees work may potentially also lean toward looseness, which could influence their desired tightness. That said, although, as demonstrated by this study, a prevention-focused individual is prone to desire tightness, it is also conceivable that the same individual working in an organization with a tight culture, located in a tight nation, may not feel the need of more tightness despite his/her focus on prevention. Considering the multilevel nature of the tightness–looseness construct, future research examining the cross-level interactions between national T-L, organizational T-L, and individual T-L would be particularly valuable in addressing this limitation.

It would also be interesting to investigate the long-term effects of employees’ desired tightness–looseness in pushing their organization in a tight or loose way. In this sense, desire for tightness (or looseness) could be considered a helpful mechanism to rebalance an extremely tight (or loose) organization and achieve tightness–looseness *ambidexterity* (Gelfand, 2018). An exceedingly tight organization has downsides, although tightness plays a critical role in the maintenance of order and coordination, which are undoubtedly essential to the organizational productivity, at the same time it lacks in fostering creativity and innovativeness. The same is also true for an extremely loose organization: having too much freedom and no coordination can only result in complete anomie and chaos. It is therefore reasonable to deduce that the employees of an excessively tight (loose) organization may feel a strong desire for looseness (tightness) which, over time, could lead to a recalibration of their organizational culture in a loose (tight) way. Future studies should implement longitudinal designs to explore this assumption deeply.

Furthermore, while our research theorizes desired tightness as a mediator, it is plausible that it may also be triggered when adverse emotional reactions to deviance rise. Given the pivotal role of prevention focus in dealing with threats and the fact that deviant organizational behaviors are perceived as threatening to organizations’ and members’ well-being, a sequential model in which work prevention focus activates desired tightness, that triggers intolerance toward deviance, which, in turn, affects again the desire for tightness, could be hypothesized. In their future works, researchers should keep in mind that the endorsement of tightness can be considered not only an activator of increased inflexibility toward norm-violating behaviors, but also an outcome.

Last but not least, within the frame of regulatory focus theory, it could be interesting to investigate the other side of the coin, the impact of the work promotion focus on the desire for looseness and, consequently, on organizational outcomes. For example, it is well-stated that prevention-focused individuals are less skilled at creative thinking in the workplace than those who are more promotion-focused (e.g., Neubert et al., 2008; Zhou et al., 2012), because creativity revolves around pushing boundaries and taking risks, mindsets that mostly belong to individuals focused on promotion. At the same time, a loose organizational culture is more likely to foster organizational creativity and innovativeness (Ozeren et al., 2013; Chua et al., 2015; Gedik and Ozbek, 2020). In this vein, a work promotion-focused employee, desirous for looseness, should be more likely to act creatively.

The present study also provides practical insights for organizations. First, because desired tightness as a result of a regulatory work prevention focus can influence employees’ reactions to norm-violating behaviors regardless of whether the behavior is detrimental or beneficial, it is crucial for leaders to recognize this trade-off. It is worth noting that, as emerged from our results, prevention-focused and tightness-hungry employees tend to react with hostility even to positive deviant behaviors, which are aimed at favoring the organization. Therefore, while it is relatively difficult for leaders to change their employees’ dispositional traits, they should be aware of the organizational climate and their personal leadership styles, as well as be able to shape them to change the attitudes and behaviors of their employees. To be more specific, according to regulatory focus theory (e.g., Higgins, 2012), promotion and prevention foci are dispositional traits, but may be also induced situationally. So that it is plausible that a certain leadership style, e.g., transformational (e.g., Delegach et al., 2017) can trigger employees’ regulatory promotion focus, that could make them wish looseness and this, in turn, could lead to greater indulgence toward positive deviant behaviors, potentially leading to benefits to the organization.

With these first results, we have begun to respond to the need for further research on the antecedents and consequences of cultural tightness in organizations, focusing on a hitherto unexplored dimension in organizational literature – the desired tightness. The T-L dimension at the individual level reveals interesting facets of employees’ perceptions and behaviors and, thus, it is deserving of further theoretical and empirical insights.

## Data availability statement

The raw data supporting the conclusions of this article will be made available by the authors, without undue reservation.

## Ethics statement

The studies involving human participants were reviewed and approved by the Department of Social and Developmental Psychological, Sapienza University of Rome. The patients/participants provided their written informed consent to participate in this study.

## Author contributions

SM collected the data and wrote the manuscript. SM and AP executed the studies and made substantial contributions to the analysis and interpretation of data. AP contributed to revising the manuscript critically for important intellectual content. All authors contributed to the article and approved the submitted version.

## Funding

This work was supported by the research funding No. AR12117A5D65E4CB (“Progetti per Avvio alla Ricerca–Tipo 1”) awarded by Sapienza University of Rome to SM.

## Conflict of interest

The authors declare that the research was conducted in the absence of any commercial or financial relationships that could be construed as a potential conflict of interest.

## Publisher’s note

All claims expressed in this article are solely those of the authors and do not necessarily represent those of their affiliated organizations, or those of the publisher, the editors and the reviewers. Any product that may be evaluated in this article, or claim that may be made by its manufacturer, is not guaranteed or endorsed by the publisher.
